# Mitochondrial metabolic reprogramming at the interface of chemoresistance and immune-cold tumor states

**DOI:** 10.3389/fimmu.2026.1872804

**Published:** 2026-07-27

**Authors:** Yanfang Liu

**Affiliations:** Department of Oncology, The Affiliated Changsha Central Hospital, Hengyang Medical School, University of South China, Changsha, China

**Keywords:** chemoresistance, immune-cold tumor, immunometabolism, mitochondrial metabolism, oxidative phosphorylation, tumor microenvironment

## Abstract

Chemoresistance and the immune-cold tumor microenvironment are major barriers to durable cancer control, yet they frequently emerge from overlapping mitochondrial adaptations selected by cytotoxic stress. In resistant tumors, mitochondria regulate oxidative phosphorylation (OXPHOS), redox buffering, apoptosis thresholds, organelle quality control, and metabolite overflow, while also shaping how antitumor immune cells experience nutrient deprivation, hypoxia, acidosis, and chronic danger signaling. Rather than assuming a single causal chain, this review distinguishes three non-equivalent relationships: direct suppression by mitochondria-derived signals, parallel emergence under shared selective pressures, and reverse causation in which immune exclusion itself facilitates mitochondrial adaptation. Within this framework, we synthesize evidence that mitochondrial adaptations support residual disease through OXPHOS dependence, mitochondrial dynamics, mitophagy, redox control, and metabolite-driven epigenetic remodeling, while concurrently reshaping T-cell, macrophage, and dendritic-cell function through lactate and acid stress, succinate, fumarate, 2-hydroxyglutarate (2-HG), adenosine, and mitochondrial DNA (mtDNA)-dependent innate immune signaling. We further highlight key determinants that influence whether mtDNA-STING signaling becomes immunogenic or suppressive, including timing, cell source, subcellular localization, and the dominant responding immune compartment. Finally, we discuss translational strategies to disrupt this mitochondria-immune interface, with emphasis on host and tumor heterogeneity, biomarker-guided selection, and treatment timing when combining with chemotherapy, mitochondrial targeting, and immunotherapy.

## Introduction

Metabolic reprogramming has moved from a descriptive hallmark of cancer to a mechanistic framework for therapeutic failure. The Warburg effect remains an important entry point, but it no longer implies that tumor mitochondria are defective or dispensable. Instead, aerobic glycolysis is best understood as a flexible metabolic state that coexists with, and often relies upon, functional mitochondrial respiration (1–5). This shift matters because chemotherapy failure often reflects not the absence of an initial cytotoxic hit, but the survival of a residual population that reorganizes its local ecology and later re-emerges as clinically resistant disease.

Mitochondria provide the bioenergetic and signaling logic for this survival state. They generate ATP through oxidative phosphorylation (OXPHOS), supply tricarboxylic acid (TCA) cycle intermediates for biosynthesis and epigenetic regulation, tune reactive oxygen species (ROS), regulate apoptosis, and store mitochondrial DNA (mtDNA) that can function as a danger signal when displaced into the cytosol or extracellular space (6–10). Chemotherapy therefore does not simply kill cells through DNA damage or mitotic stress. It also selects for mitochondrial fitness, favoring cells that can rewire respiration, redox set points, mitophagy, organelle dynamics, and stromal communication.

The immune consequence of this mitochondrial rewiring is often discussed too broadly. Tumors labeled as “immune cold” are commonly defined by poor cytotoxic T-cell infiltration, weak antigen presentation, suppressive myeloid or fibroblast barriers, low interferon activity, and limited response to immune checkpoint blockade. Yet coldness is not merely a static histologic category, nor is it a single biology. Recent work emphasizes at least three non-equivalent topologies: immune desert, immune excluded, and immune suppressed states, each with distinct spatial and metabolic constraints (11–16). A tumor that consumes oxygen, glucose, glutamine, and amino acids while releasing lactate, succinate, fumarate, 2-HG, adenosine, oxidized mtDNA, and inflammatory mediators may not suppress all of these states in the same way.

This distinction is essential for mechanistic interpretation. Rather than treating mitochondrial reprogramming as a universally proven driving force, we evaluate where evidence supports direct causation, where mitochondrial and immune phenotypes likely co-emerge under shared selective pressures, and where reverse causation remains plausible because immune exclusion itself reduces metabolic competition and permits tumor mitochondrial adaptation. The phrase “mitochondria-centered loop” is therefore used as a heuristic model rather than as a claim that every chemoresistant tumor follows one deterministic sequence.

**Figure 1:**
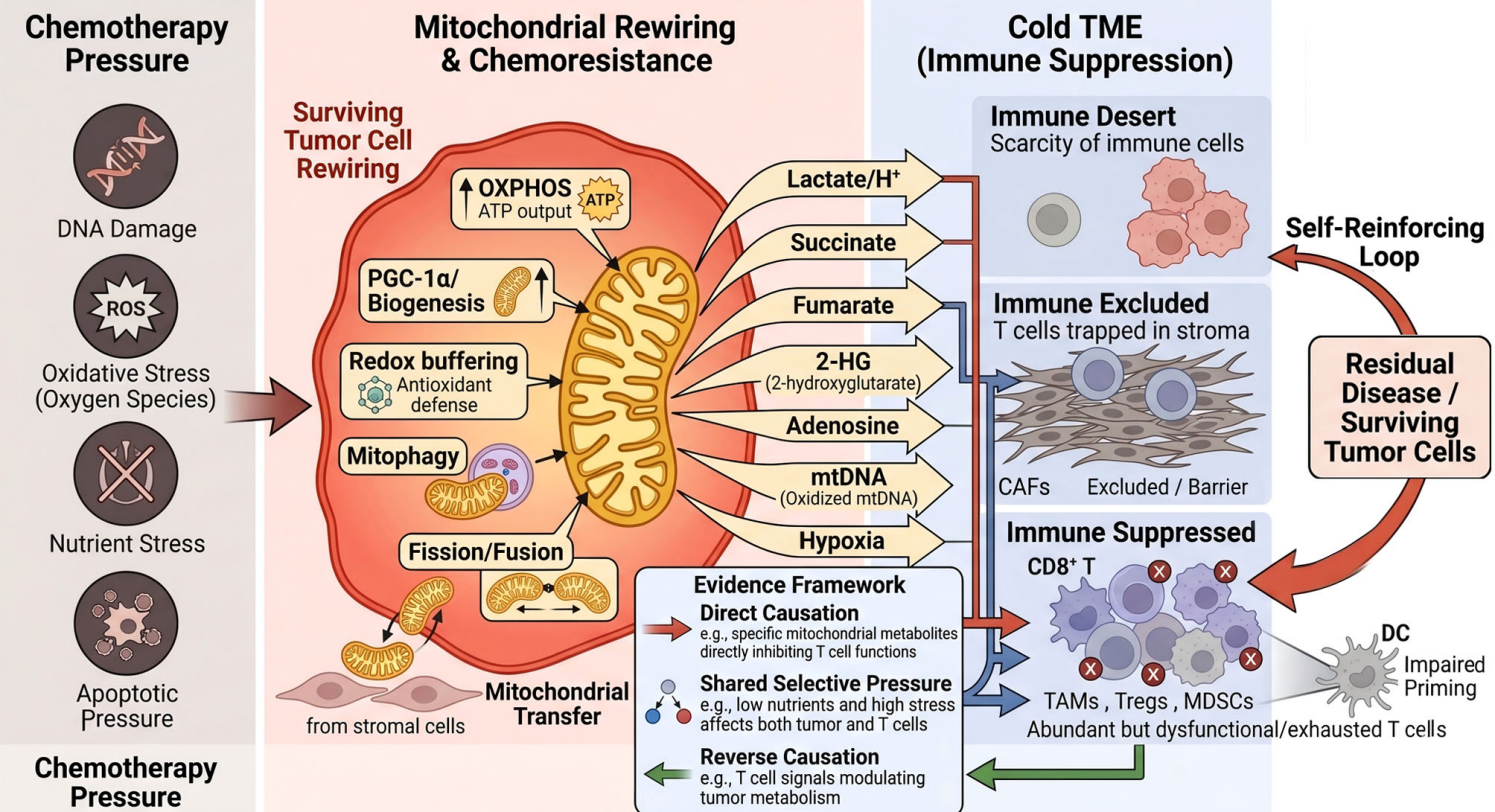
Chemotherapy pressure selects mitochondrial rewiring programs that support chemoresistance and contribute to immune-cold tumor microenvironmental states. DNA damage, oxidative stress, nutrient stress, and apoptotic pressure enrich surviving tumor cells with enhanced oxidative phosphorylation (OXPHOS) and ATP output, PGC-1alpha-associated mitochondrial biogenesis, redox buffering, mitophagy, fission/fusion dynamics, and mitochondrial transfer from stromal cells. These adaptations promote residual disease while increasing the release or accumulation of lactate/H+, succinate, fumarate, 2-hydroxyglutarate (2-HG), adenosine, oxidized mitochondrial DNA (mtDNA), and hypoxia-related signals. Together, these mitochondrial outputs are associated with distinct cold tumor microenvironment (TME) topologies, including immune desert, immune-excluded, and immune-suppressed states, characterized by scarcity of immune cells, cancer-associated fibroblast (CAF)-mediated stromal barriers, dysfunctional or exhausted CD8+ T cells, enrichment of tumor-associated macrophages (TAMs), regulatory T cells (Tregs), and myeloid-derived suppressor cells (MDSCs), as well as impaired dendritic-cell (DC) priming. The evidence framework shown in the figure emphasizes that individual mechanisms may operate through direct causation, shared selective pressure, or reverse causation, thereby supporting a self-reinforcing loop between mitochondrial adaptation, chemoresistance, and maintenance of the cold TME.

Against this background, this review has three aims. First, it summarizes how mitochondrial OXPHOS, redox buffering, organelle dynamics, mitophagy, and mitochondrial transfer support resistance to cytotoxic therapy. Second, it examines how mitochondrial outputs reshape antitumor immunity through nutrient competition, lactate and acid stress, macrophage and dendritic-cell rewiring, TCA-cycle metabolites, hypoxia-adenosine signaling, and mtDNA-dependent innate immune sensing. Third, it discusses how biomarker-guided targeting of these processes might convert residual disease from an immune-cold state into a more immune-visible one. Within this framework, the key question is not whether mitochondria matter, but when they act as direct effectors, when they act as amplifiers, and when they mainly mark a resistant ecological state (**Figure 1**).

## Mitochondrial metabolic plasticity under chemotherapy pressure

Counterintuitively, across multiple tumor models, cells that survive therapy often become more, not less, dependent on mitochondrial respiration. Yet the pathways that feed this state are not identical across tumors. In pancreatic cancer, cells that persist after oncogene ablation require mitochondrial function, identifying OXPHOS as a survival program for residual disease (17). Complementing this, glycolytic pancreatic cancer models exposed to LDHA inhibition can escape through AMPK-mTOR-S6K-driven augmentation of OXPHOS, illustrating how glycolytic pressure can reroute metabolism toward respiratory survival (18). In acute myeloid leukemia (AML), cytarabine-resistant human cells adopt a high-mitochondrial-potential oxidative state rather than a purely glycolytic phenotype (19). In colorectal cancer, chemotherapy induces a SIRT1/PGC-1alpha-dependent increase in OXPHOS and mitochondrial biogenesis that protects patient-derived cultures and xenografts (20). These findings converge on a clinically relevant principle: residual cancer cells can exploit mitochondrial plasticity to enter and maintain a drug-tolerant state, but they achieve this through context-specific fuel rerouting, mitochondrial biogenesis, and oxidative stress adaptation rather than through one universal pathway.

Whether this plasticity protects or sensitizes tumor cells is context-dependent. In ovarian cancer, PML-regulated mitochondrial metabolism can enhances chemosensitivity, showing that mitochondrial activity may either shield or expose a tumor depending on how apoptosis and stress responses are wired (21). More recently, NIT2 was shown to restrain OXPHOS and enhance chemosensitivity in gastric cancer, reinforcing that mitochondrial respiration is not uniformly beneficial to cancer cells, but becomes a selectable trait under defined genetic and therapeutic conditions (22). This duality matters therapeutically. A blanket assumption that all OXPHOS-high tumors should be treated identically may fail unless paired with biomarkers of respiratory dependence, redox buffering capacity, substrate use, and apoptotic competence.

**Figure 2:**
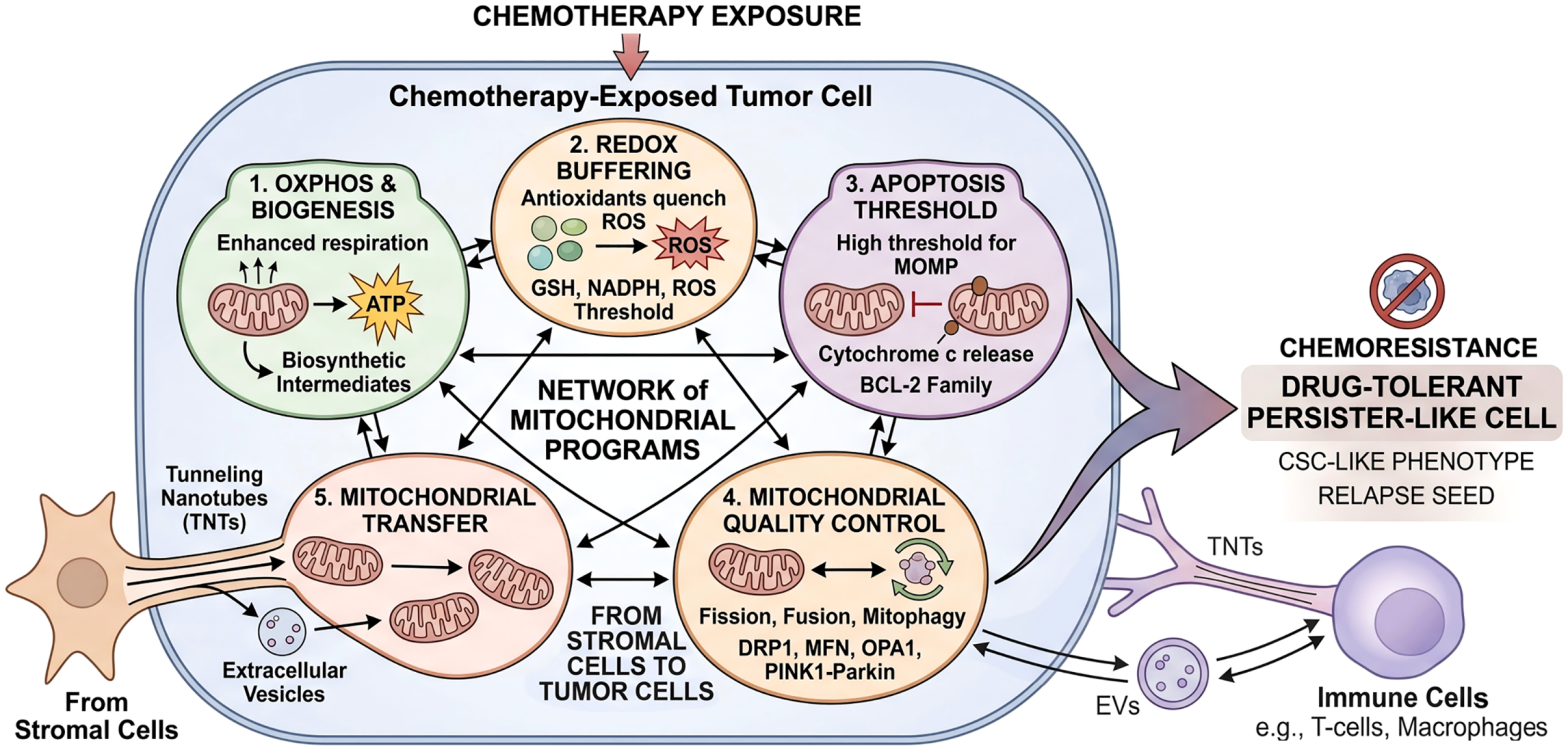
Tumor cell-intrinsic mitochondrial programs that support chemotherapy tolerance and chemoresistance. Upon chemotherapy exposure, surviving tumor cells engage a network of mitochondrial adaptations composed of five interconnected modules: (1) enhanced oxidative phosphorylation (OXPHOS) and mitochondrial biogenesis, which sustain ATP production and biosynthetic intermediates; (2) redox buffering, in which antioxidant systems maintain glutathione (GSH), NADPH, and reactive oxygen species (ROS) below lethal thresholds; (3) elevation of the apoptotic threshold, including restraint of mitochondrial outer membrane permeabilization (MOMP), reduced cytochrome c release, and BCL-2 family-dependent survival signaling; (4) mitochondrial quality control through fission, fusion, and mitophagy, involving regulators such as DRP1, MFN, OPA1, and PINK1-Parkin; and (5) mitochondrial transfer to tumor cells through tunneling nanotubes (TNTs) or extracellular vesicles (EVs), particularly from stromal cells. Together, these coordinated mitochondrial programs support chemoresistance and favor the emergence of drug-tolerant persister-like cells, cancer stem cell-like phenotypes, and relapse-seeding tumor cell populations.

Mitochondrial translation and respiratory-chain assembly provide additional vulnerabilities. In AML, inhibition of mitochondrial translation has been proposed as a therapeutic strategy, exploiting the high mitochondrial dependence of malignant hematopoietic cells (23). Direct complex I inhibition likewise demonstrated that OXPHOS suppression can uncover cancer vulnerability, but translation into the clinic has been constrained by a narrow therapeutic window. In phase I testing, IACS-010759 showed limited efficacy and dose-limiting lactate elevation and neurotoxicity, indicating that OXPHOS inhibitors will likely require stricter biomarker selection, schedule control, and tissue-safety monitoring (24). Strategies that reduce oxygen consumption may still be useful in selected combination settings, particularly when the goal is to relieve hypoxia rather than simply maximize tumoricidal OXPHOS blockade (25). Together these studies indicate that mitochondrial metabolism is not merely an adaptive consequence of chemotherapy; in selected contexts, it becomes a therapeutically actionable vulnerability of the chemoresistant state (**Figure 2**).

## Redox buffering, apoptotic thresholds, and mitochondrial quality control

Chemotherapy frequently kills through DNA damage, replication catastrophe, mitotic stress, and induction of mitochondrial apoptosis. Mitochondrial reprogramming can blunt these effects by raising the apoptotic threshold and reinforcing mitochondrial quality control (26–29). Respiratory adaptationcan sustain ATP-dependent repair and drug-efflux programs, while antioxidant and mitophagic circuits restrain mitochondrial ROS from crossing the lethal threshold (10, 26, 29). This is clinically consequential because moderate ROS can support signaling, proliferation, and stress adaptation, whereas excessive ROS drives macromolecular damage, mitochondrial outer membrane permeabilization (MOMP), and cell death (27, 29). The chemoresistant cell therefore does not simply reduce oxidative stress; it tunes redox intensity so that ROS becomes a messenger rather than an executioner.

Mitochondrial quality control helps maintain this redox window. CRL4 targeting in ovarian cancer suppresses chemoresistant growth by inducing mitophagy, illustrating how mitophagy can be manipulated to destabilize resistant cells (26). More broadly, mitochondrial dynamics influence the response to chemotherapy by controlling fission, fusion, cristae architecture, cytochrome c release, calcium buffering, and organelle distribution (30). The mitophagy machinery, including PINK1-Parkin-dependent pathways and receptor-mediated mechanisms, removes damaged mitochondria and prevents accumulation of excessive ROS or immunostimulatory mtDNA (28). In resistant tumors, this cleaning system can be co-opted to preserve a fit mitochondrial network during repeated cytotoxic injury.

Mitochondrial dynamics add another layer of adaptation. Fission can distribute mitochondria to regions of high energy demand, support migration and invasion, and segregate damaged organelles for mitophagy; fusion can dilute localized damage, maintain mitochondrial membrane potential, and support stress tolerance (30). Mitochondrial transfer between stromal and cancer cells may further rescue bioenergetic defects or replenish damaged organelles after therapy (31). Importantly, transfer is not restricted to stromal rescue of tumor cells. Intercellular nanotubes can mediate mitochondrial trafficking between cancer and immune cells, and more recent work suggests that tumor-associated mitochondrial transfer can actively impair T-cell function and promote immune evasion (32–34). These processes suggest that the resistant state is not defined only by gene expression. It is also defined by organelle ecology: the capacity to remodel, remove, replace, export, and sometimes import mitochondria under therapeutic stress.

## From mitochondrial fitness to an immune-cold microenvironment

Immune-cold tumors were initially described in spatial and cellular terms: poor T-cell infiltration, immune exclusion at the invasive margin, dense stromal barriers, dysfunctional antigen presentation, and low productive interferon signaling (11–14). Tumor-intrinsic programs can enforce this topology. For example, a melanoma cell program promotes T-cell exclusion and resistance to checkpoint blockade, and cancer-associated fibroblast matrix programs can spatially restrict T cells in lung tumors (35, 36). Pan-cancer immune profiling has further demonstrated that immune landscapes vary widely across malignancies, supporting the concept that immune coldness is a biological state rather than simply a low lymphocyte count (37).

However, the term “immune cold” should not be used as if it described one uniform microenvironment. Recent consensus work distinguishes at least immune desert, immune excluded, and immune suppressed states (15, 16). These topologies likely differ in their dominant mitochondrial determinants. Respiratory oxygen consumption, fibroblast-supported acid gradients, and lactate accumulation may preferentially reinforce exclusion; defective antigen presentation, cDC1 scarcity, and myeloid dominance may sustain desert states; and persistent adenosine, mtDNA-linked innate signaling, or metabolite stress may trap infiltrating T cells in dysfunctional or terminally exhausted programs. This distinction matters mechanistically because a therapy that improves T-cell entry is not necessarily the same therapy that restores dendritic-cell priming or rescues exhausted T cells.

Mitochondrial metabolism helps generate these states by imposing energetic competition, but the causal direction is not identical across mechanisms. Cancer cells with high respiratory and anabolic demand consume glucose, oxygen, amino acids, and lipid substrates that immune cells also require for activation and effector function. Metabolic competition in the tumor microenvironment can drive cancer progression, while restoration of phosphoenolpyruvate metabolism improves antitumor T-cell responses (38, 39). T cells need coordinated glycolysis and mitochondrial fitness to proliferate, produce cytokines, develop memory, and maintain cytotoxicity (40). When the microenvironment restricts substrates and oxygen, T cells cannot simply compensate by switching fuel. Their mitochondria become a bottleneck. At the same time, some associations remain inferential rather than fully causal. For example, OXPHOS-high residual disease may coexist with T-cell exclusion because both are selected by hypoxia, stromal structure, or post-chemotherapy remodeling rather than because one uniformly causes the other.

This bottleneck is especially important when distinguishing T-cell states. “Exhaustion, “ “dysfunction, “ and “exclusion” are related but not interchangeable. Progenitor-exhausted TCF1+ CD8+ T cells retain proliferative potential and can respond to checkpoint blockade, whereas terminally exhausted PD-1hi TOXhi cells display more severe mitochondrial insufficiency, fixed epigenetic programs, and limited reinvigoration potential (41–44). Continuous antigenic stimulation under hypoxia can rapidly accelerate mitochondrial stress and exhaustion programs (45). Accordingly, a metabolically hostile microenvironment may not only reduce T-cell abundance, but also determine which exhausted state predominates. This is one reason why immune-cold tumors with abundant but metabolically trapped T cells should not be conflated with true immune deserts.

Myeloid cells are equally important in this transition. Macrophage polarization is not explained by lactate exposure alone. Tumor cells and macrophages compete for glutamine, fatty acids, and other carbon sources, while macrophage mitochondrial dynamics influence inflammatory versus reparative programs (46, 47). Mitochondrial fission and fusion help determine whether macrophages sustain inflammatory stress or adopt more survival-oriented, M2-like states (48). In parallel, macrophage-rich spatial niches formed after chemotherapy can confine exhausted T cells without proving that tumor mitochondrial reprogramming is the sole initiating event (49). We therefore interpret such studies as strong ecological support for the loop model, while explicitly recognizing that the direct mechanistic bridge between chemotherapy-selected tumor mitochondria and the final immune topology remains incomplete in some settings.

Dendritic-cell biology provides a further layer of specificity. Conventional type 1 dendritic cells (cDC1s) are critical for cross-presentation and priming of tumor-reactive CD8+ T cells (50). Their function can be constrained by the same metabolic landscape that selects chemoresistant tumor cells. Succinate, itaconate-linked macrophage programs, and chronic myeloid suppression can blunt effective cross-talk between innate sensing and adaptive priming, while recent work indicates that mitochondrial metabolism and signaling directly shape dendritic-cell function in antitumor immunity (51, 52). This is particularly relevant for the current topic because mitochondrial danger signals are clinically meaningful only if they are converted into productive dendritic-cell activation rather than captured by suppressive myeloid circuits.

Chemotherapy can intensify all of these paradoxes. Effective cytotoxic therapy should release antigens and danger signals, yet it can also produce myeloid-rich spatial niches that confine exhausted T cells, as shown in ovarian cancer (49). Additional metabolic pressures, including cholesterol accumulation and ammonia, can deepen T-cell dysfunction (43, 53, 54). Thus, after chemotherapy, the tumor bed may contain both more tumor antigen and less functional immunity. In this sense, the mitochondrial immune-cold loop is best understood as a context-dependent ecological model: mitochondrial adaptations can act as direct suppressors in some mechanisms, as amplifiers of pre-existing stromal or myeloid barriers in others, and as markers of shared post-therapy selection in still others.

## Lactate overflow and acid stress as immune silencers

Lactate is often treated as a glycolytic byproduct, but its immunologic effects are inseparable from mitochondrial state. Mitochondrial respiration, hypoxia, pyruvate handling, NAD+/NADH balance, and monocarboxylate transporter activity determine whether carbon flows into oxidation, secretion, or exchange. Tumor-derived lactic acid directly inhibits human T cells, reducing proliferation and cytokine production (55). It also polarizes tumor-associated macrophages toward phenotypes that promote tumor progression (56). In melanoma models, LDHA-associated lactic acid production blunts T-cell and NK-cell immunosurveillance, linking lactate accumulation to escape from cytotoxic immunity (57).

Mechanistically, it is important to distinguish lactate anion from proton-associated acid stress rather than treating “lactate” as a single signal. As emphasized by more recent syntheses, intracellular acidification is often a dominant suppressor of CD8+ T-cell effector function, whereas lactate itself can serve as a fuel or signaling metabolite in more adaptable immune subsets such as tumor-adapted Tregs and selected macrophage states (58–60). This distinction helps explain why some intratumoral immune cells are inhibited while others are sustained. Lactate exposure is also spatially heterogeneous because regional perfusion, oxygenation, vessel distance, and the distribution of MCT4-high glycolytic versus MCT1-high oxidative compartments shape local lactate production, exchange, and clearance. Accordingly, immune cells in hypoperfused acidic regions and better-perfused niches encounter different combinations of lactate, protons, oxygen, and glucose, which can produce divergent functional effects (58, 61).

Regulatory T cells (Tregs) can exploit this hostile lactate-rich niche. Tumor-infiltrating Tregs use lactic acid as metabolic support, and lactic acid can promote PD-1 expression in Tregs within highly glycolytic tumor microenvironments (59, 60). This creates a metabolic asymmetry: cytotoxic T cells and NK cells are suppressed by acidic lactate stress, whereas suppressive Tregs and some macrophage states remain adapted or even advantaged. The result is not simply immune inhibition, but immune sorting. The metabolic microenvironment selects which immune cells can remain functional, and may therefore influence whether a tumor becomes predominantly immune excluded or immune suppressed rather than simply “cold.”

Lactate also modifies gene regulation. Histone lactylation provides a direct link between metabolite accumulation and transcriptional reprogramming, and glucose-driven histone lactylation promotes the immunosuppressive activity of monocyte-derived macrophages in glioblastoma (62, 63). Mechanistically, intracellular lactate-driven histone lactylation increased IL-10 expression in monocyte-derived macrophages, and this IL-10-centered program was required for suppression of T-cell activity (62). These findings suggest that lactate is not only a marker of glycolytic flux; it is an epigenetic substrate that can stabilize suppressive immune states. In this framework, macrophages respond not only to extracellular acidity but also to lactate-dependent transcriptional remodeling, which may help sustain tumor-associated macrophage (TAM) programs after chemotherapy.

**Figure 3:**
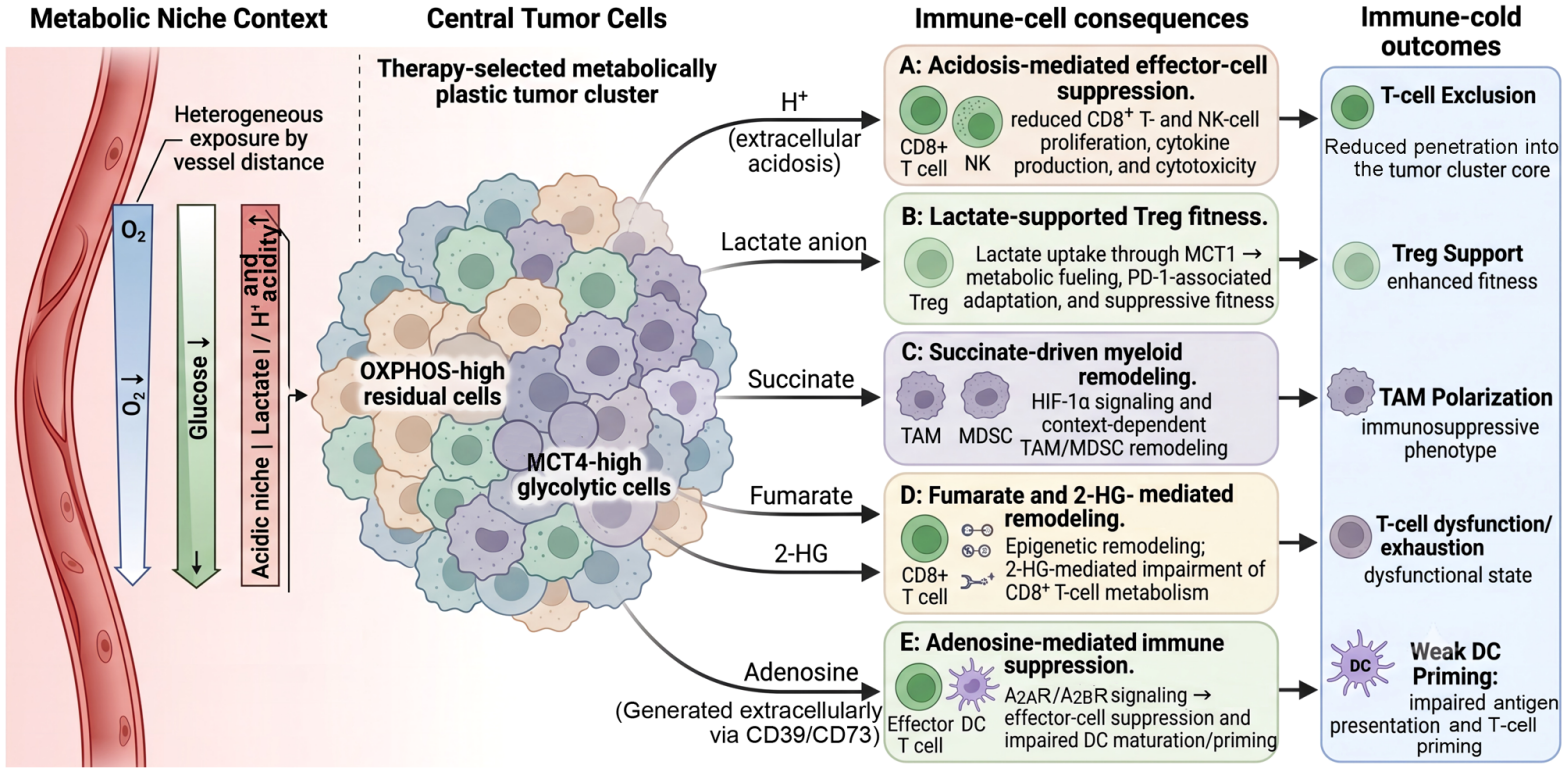
Spatial metabolic niches promote immune-cold tumor outcomes. Increasing vessel distance reduces oxygen and glucose availability while increasing lactate, H^+^, and acidity. OXPHOS-high residual cells coexist with MCT4-high glycolytic cells. Acidosis suppresses CD8^+^ T- and NK-cell function, whereas MCT1-dependent lactate uptake supports Treg fitness. Succinate promotes context-dependent myeloid remodeling, while fumarate and 2-hydroxyglutarate (2-HG) induce epigenetic and T-cell metabolic dysfunction. CD39/CD73-generated adenosine further suppresses effector immunity and DC priming. Together, these mechanisms promote T-cell exclusion and dysfunction, regulatory T-cell (Treg) support, tumor-associated macrophage (TAM) polarization, and an immune-cold microenvironment.

Monocarboxylate transporters (MCTs) organize this lactate economy by controlling export, import, and metabolic symbiosis between cancer cells and stromal or immune cells (61). MCT1-dependent lactate uptake appears particularly relevant for tumor-adapted Tregs, whereas tumor and stromal compartments often rely on coordinated MCT1/MCT4 exchange to maintain lactate flux (58, 59, 61). Consequently, MCT blockade, LDHA inhibition, buffering of acidity, and normalization of mitochondrial pyruvate oxidation are not interchangeable strategies. Each targets a different level of the lactate circuit and may have different consequences for tumor cells, Tregs, macrophages, and effector lymphocytes. A rational anti-cold strategy should therefore ask whether lactate is acting primarily as an acid stressor, a carbon fuel, an epigenetic substrate, or a spatial cue that partitions immune-cell states (**Figure 3**).

## TCA-cycle metabolites and hypoxia-adenosine signaling

Mitochondrial metabolites are increasingly recognized as immune instructions rather than passive byproducts. Succinate can stabilize HIF-1alpha and induce IL-1beta in inflammatory macrophages (64). In tumors with succinate dehydrogenase dysfunction, succinate links TCA-cycle disruption to pseudohypoxia by inhibiting HIF-alpha prolyl hydroxylases (65). Fumarate and succinate can inhibit alpha-ketoglutarate-dependent histone and DNA demethylases, connecting mitochondrial dysfunction to epigenetic persistence (66). These mechanisms are relevant to immune coldness because HIF signaling, epigenetic reprogramming, and chronic inflammatory cues all shape antigen presentation, myeloid differentiation, stromal remodeling, and T-cell exclusion.

One gap in many simplified TCA-cycle narratives is the relative absence of itaconate. In inflammatory macrophages, itaconate links mitochondrial metabolism to succinate dehydrogenase inhibition, metabolic remodeling, and dampening of inflammatory output (67, 68). This matters in tumors because immunosuppressive myeloid states are not defined only by succinate accumulation or lactate exposure; they also involve itaconate-centered rewiring that can restrain dendritic-cell function and adaptive priming. Indeed, recent work shows that macrophage-derived itaconate can suppress dendritic-cell activity and promote acquired resistance to anti-PD-1 therapy (51). Therefore, a mitochondria-centered model of immune coldness should incorporate not only tumor-derived metabolites, but also suppressive metabolites generated by myeloid cells in response to chronic tumor stress.

The IDH-mutant oncometabolite 2-HG provides a particularly clear example of metabolite-driven immune suppression. Cancer-associated IDH mutations produce 2-HG (69). In gliomas, mutant IDH1 regulates the tumor-associated immune system (70), and the R enantiomer of 2-HG suppresses antitumor T-cell immunity (71). More recent work shows that D-2HG can directly alter T-cell metabolism and impair CD8+ T-cell function (72). These studies demonstrate that mitochondrial or mitochondria-adjacent metabolic lesions do not merely support cancer-cell growth; they can set the immune tone of an entire tissue.

Succinate biology also illustrates why this field must avoid one-directional conclusions. Cancer-derived succinate can promote macrophage polarization and metastasis through the succinate receptor (73). Yet succinate can also preserve CD8+ T-cell fitness and augment antitumor immunity under specific experimental conditions (74). These scenarios are mechanistically distinct. Exogenous succinate supplementation *in vitro* or adoptive-transfer models should not be conflated with the concentration, compartmentalization, and receptor context of tumor-derived succinate *in situ*. A metabolite’s immune meaning depends on its source, subcellular localization, uptake route, receptor expression, redox context, oxygen tension, and timing. Therapeutically, the goal should be to reconfigure harmful metabolite gradients rather than simply label a molecule as pro- or anti-tumor.

Hypoxia-adenosine signaling is another metabolic bridge between mitochondrial demand and immune coldness. Tumor respiration and abnormal vasculature create oxygen gradients that stabilize HIF-1alpha, promote myeloid-derived suppressor-cell function, and increase PD-L1 expression under hypoxia (75, 76). Hypoxia and treatment-related cell damage enrich extracellular ATP; CD39 hydrolyzes ATP and ADP to AMP, and CD73 converts AMP to adenosine, while HIF-dependent reinforcement of the ectonucleotidase program further favors adenosine accumulation (77–79). A2A and A2B receptors are Gs-coupled receptors that increase intracellular cAMP-PKA signaling. In CD8+ T cells and NK cells, this signaling attenuates T-cell receptor or activating-receptor signaling, IFN-gamma production, and cytotoxicity; in parallel, adenosine supports Treg suppressive activity, MDSC and tumor-associated macrophage programs, and dendritic-cell states with weaker maturation and cross-priming (77–79). Thus, mitochondrial oxygen consumption can indirectly organize immune access and function by linking local hypoxia to an enzymatic adenosine-generating circuit and receptor-mediated immune suppression.

## Mitochondrial danger signaling: immunogenic spark or chronic immune coldness

Mitochondrial danger signaling is a double-edged process. mtDNA retains bacterial-like features and can activate innate immune sensors when released from mitochondria. Mitochondrial DNA stress primes antiviral innate immunity (80). During apoptosis, BAK/BAX macropores can facilitate mitochondrial herniation and mtDNA efflux, while mitochondrial inner membrane permeabilisation enables mtDNA release and cGAS-STING activation when caspases are inhibited or bypassed (81, 82). Oxidized mtDNA fragments can exit through mPTP- and VDAC-dependent channels to activate NLRP3 inflammasome and interferon signaling (83). These pathways explain why mitochondrial injury can make cell death immunologically potent.

However, danger signaling is beneficial only when it is acute, localized, and coupled to antigen presentation. Persistent or spatially misdirected mtDNA signaling may instead reinforce suppression. Fumarate can induce vesicular release of mtDNA to drive innate immunity (84), and chromosomal instability can promote metastasis through cytosolic DNA signaling (85). Direct STING activation in the tumor microenvironment can produce systemic tumor regression in preclinical models (86), consistent with the idea that tumor-cell DNA signals can be immunogenic when dendritic-cell priming is preserved (50). By contrast, mtDNA released by senescent tumor cells can be taken up by polymorphonuclear myeloid-derived suppressor cells (PMN-MDSCs), activating cGAS-STING-NF-kB signaling and amplifying suppressive myeloid activity (87).

The key determinants of this switch deserve explicit emphasis. First, timing matters: acute mitochondrial injury may cooperate with immunogenic cell death and type I interferon priming, whereas chronic release from stressed or senescent cells can sustain suppressive inflammation. Second, the source cell matters: tumor-cell mtDNA, myeloid-cell mtDNA handling, and stromal uptake do not produce equivalent downstream programs. Third, subcellular and extracellular localization matters: cytosolic sensing, endosomal uptake, and vesicular transfer can engage different sensors and trafficking routes. Fourth, the metabolic state of the receiving compartment matters: cDC1s, macrophages, and exhausted T cells interpret the same signal through very different signaling and bioenergetic constraints (50, 52). In practical terms, oxidized mtDNA in macrophage-lineage cells can drive inflammasome and interferon programs (83), whereas dendritic cells require a permissive mitochondrial state to translate innate sensing into productive antitumor priming (52). Therefore, the mtDNA-STING axis should be viewed less as a binary on/off switch and more as a context-dependent signaling grammar.

**Figure 4:**
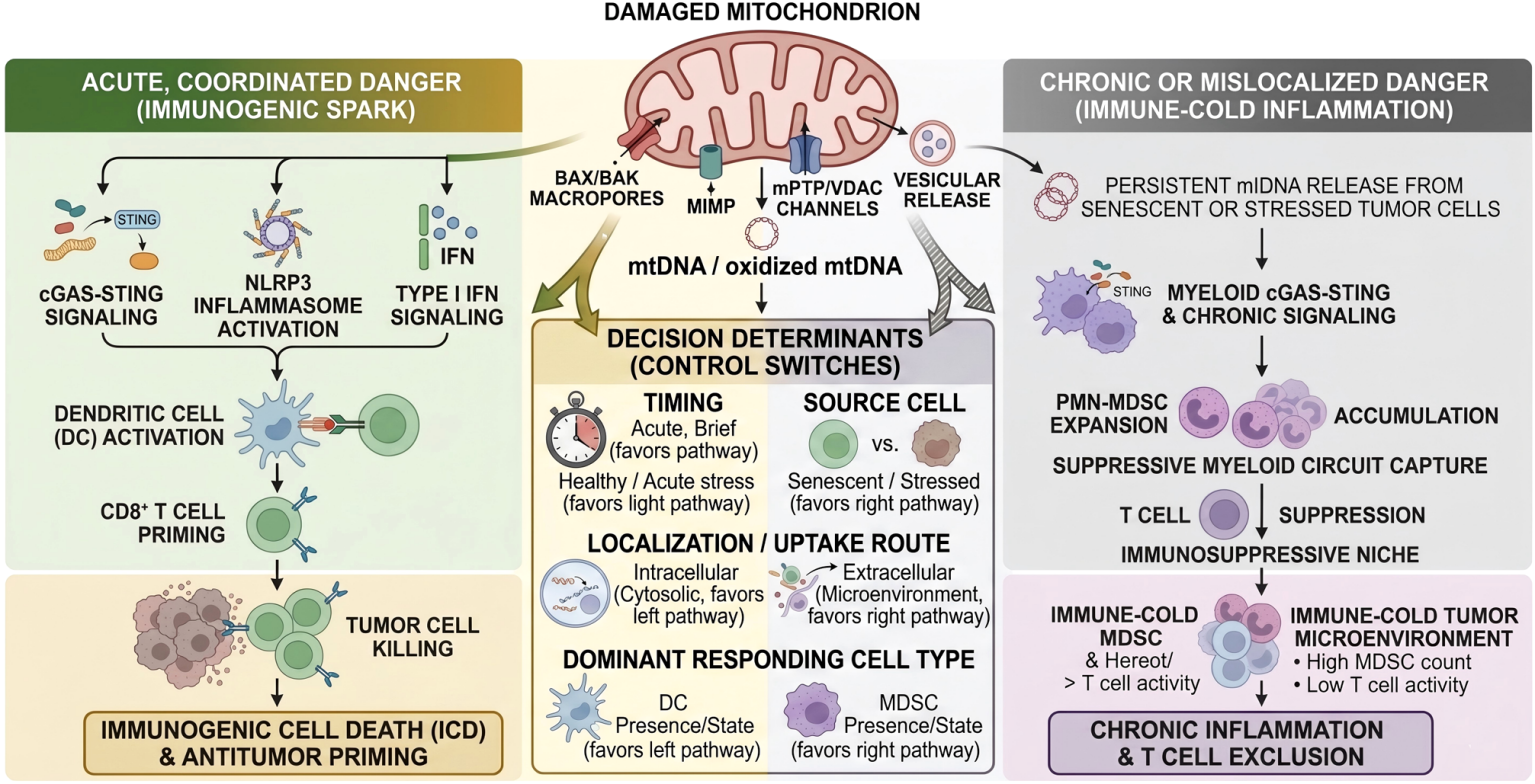
Mitochondrial danger signaling can generate either immunogenic priming or immune-cold inflammation, depending on release context and the dominant responding cell type. Damaged mitochondria may release mitochondrial DNA (mtDNA) or oxidized mtDNA through BAX/BAK macropores, mitochondrial inner membrane permeabilization (MIMP), mPTP/VDAC-associated channels, or vesicular pathways. When these signals arise acutely and in a coordinated manner, they can activate cGAS-STING signaling, the NLRP3 inflammasome, and type I interferon (IFN) programs, thereby promoting dendritic-cell (DC) activation, CD8+ T-cell priming, tumor cell killing, and immunogenic cell death (ICD). In contrast, persistent or mislocalized mtDNA release from senescent or stressed tumor cells may preferentially engage myeloid cGAS-STING signaling and chronic inflammatory circuits, leading to PMN-MDSC expansion, suppressive myeloid circuit capture, T-cell suppression, and formation of an immune-cold tumor microenvironment. The figure highlights four major control switches that shape this bifurcation: timing, source cell, localization or uptake route, and the dominant responding cell type. Together, these determinants influence whether mitochondrial danger signals support antitumor immunity or reinforce chronic inflammation and T-cell exclusion.

This duality is central to chemotherapy. Many cytotoxic drugs induce mitochondrial stress, apoptosis, senescence, or sublethal organelle injury. If danger signals are synchronized with dendritic-cell uptake, type I interferon production, and T-cell priming, they can promote immune-mediated clearance. If they persist after antigen presentation fails, or if they are captured by suppressive myeloid cells, they may help create a chronically inflamed but immune-cold niche. The clinically relevant question is therefore not whether chemotherapy induces mitochondrial danger, but whether that danger is interpreted as a vaccination signal or as tissue damage requiring suppression (**Figure 4**).

## Therapeutic strategies to break the mitochondrial immune-cold loop

Mitochondria-centered therapy should not be reduced to maximal OXPHOS inhibition. Mitochondria are essential in cardiac, neural, hematopoietic, and immune tissues, and effector T cells also require mitochondrial fitness for proliferation, persistence, and recovery from exhaustion. The more realistic goal is selective pressure reversal: identify tumors in which chemotherapy has selected an OXPHOS-high, redox-buffered, immune-cold residual state, then target the tumor mitochondrial dependency while preserving or restoring immune-cell metabolism. This requires biomarkers such as mitochondrial mass, respiratory-chain gene expression, oxygen consumption, lactate gradients, mtDNA release signatures, hypoxia-adenosine markers, and spatial immune mapping.

The first therapeutic axis is direct targeting of mitochondrial respiration and translation. Complex I inhibitors, mitochondrial translation inhibitors, and OXPHOS-targeting drugs may sensitize resistant cancer cells when respiratory dependence is high (23–25, 88). Yet the IACS-010759 experience shows that on-target complex I inhibition does not automatically translate into a wide therapeutic window, and tissue toxicity remains a central limitation (24). In some tumor contexts, respiratory activity remains linked to therapy sensitivity rather than resistance, and in T cells mitochondrial support may be required for effective reinvigoration. This context dependence argues against treating high OXPHOS as a universal liability without spatial and cell-type resolution.

Clinical development of mitochondrial inhibitors further illustrates this point. Devimistat (CPI-613), which targets pyruvate dehydrogenase and alpha-ketoglutarate dehydrogenase-dependent mitochondrial metabolism, showed encouraging activity in combination with cytarabine and mitoxantrone in a phase II study of acute myeloid leukemia (89). By contrast, the phase III AVENGER 500 study failed to improve outcomes when devimistat was added to modified FFX in metastatic pancreatic adenocarcinoma (90). These results do not negate mitochondrial targeting; rather, they underscore the importance of disease context, combination partner, biomarker selection, and metabolic state at the time of treatment. Atovaquone, by inhibiting mitochondrial respiration and alleviating tumor hypoxia, provides a complementary example in which reducing oxygen consumption may improve radiosensitivity and potentially reshape immune access (91).

The second axis is immunogenic cell death (ICD). Classic ICD involves calreticulin exposure, ATP release, HMGB1 release, autophagy-dependent antigen handling, dendritic cell activation, and T cell priming (92–96). Mitochondrial stress can amplify or distort these signals. A future chemotherapy-immunotherapy combination should therefore be judged not only by tumor shrinkage, but by whether it produces immunogenic mitochondrial injury. For example, a drug that increases mtDNA sensing without restoring antigen presentation may not heat a cold tumor; it may only create suppressive inflammation.

The third axis is metabolic normalization of the immune microenvironment. LDHA or MCT inhibition, buffering of tumor acidity, targeting adenosine-generating enzymes or receptors, modulation of HIF-1alpha signaling, and interruption of succinate-, itaconate-, or 2-HG-centered suppressive circuits may reduce the metabolic barrier that excludes T cells or traps them in dysfunctional states. These interventions are likely to work best when combined with chemotherapy-induced antigen release and immune checkpoint blockade. The ideal sequence may be: debulk and expose antigen, reduce metabolite-mediated suppression, then sustain T-cell function through checkpoint blockade and mitochondrial support.

The fourth axis is immune-cell mitochondrial rescue. Mitochondrial activation chemicals can synergize with PD-1 blockade for T-cell-dependent antitumor activity (97). Metformin can promote antitumor immunity by inducing endoplasmic-reticulum-associated degradation of PD-L1 (98), while also inhibiting mitochondrial complex I in cancer cells (88). These findings illustrate a useful principle: the same metabolic drug may act on both tumor cells and immune cells, but the direction of benefit depends on dose, timing, tissue distribution, and baseline mitochondrial state. Future trials should therefore collect immune-metabolic pharmacodynamic data rather than treating metabolism as an invisible background variable.

**Figure 5:**
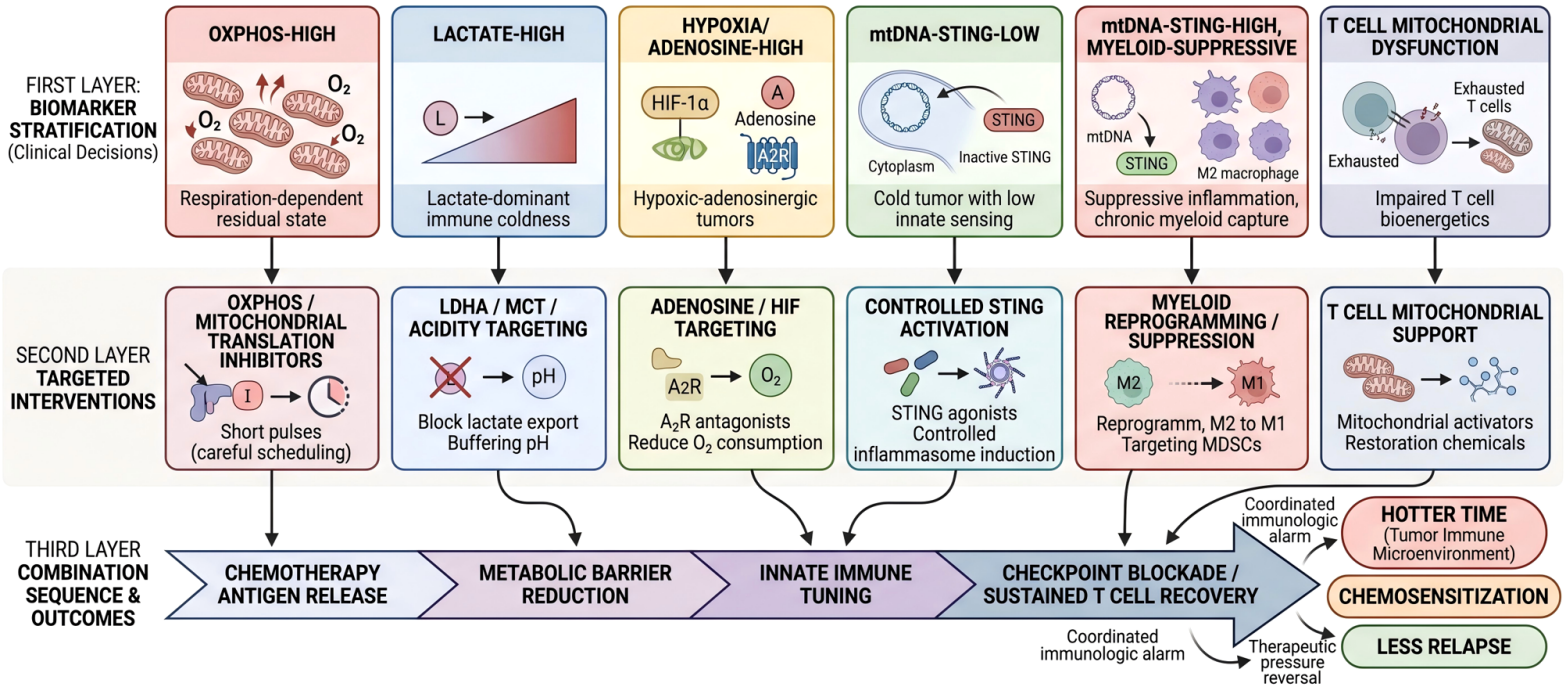
Therapeutic roadmap for converting mitochondrial stress into immune activation. This schematic is organized into three layers: biomarker stratification, targeted interventions, and combination sequence/outcomes. The upper layer identifies clinically relevant mitochondrial-immune states, including OXPHOS-high residual disease, lactate-high immune-cold tumors, hypoxia/adenosine-high tumors, mtDNA-STING-low tumors, mtDNA-STING-high myeloid-suppressive tumors, and T-cell mitochondrial dysfunction. The middle layer outlines corresponding interventions, including OXPHOS or mitochondrial translation inhibitors, LDHA/MCT/acidity targeting, adenosine/HIF targeting, controlled STING activation, myeloid reprogramming, and T-cell mitochondrial support. The lower layer emphasizes biomarker-guided therapeutic sequencing, in which chemotherapy-induced antigen release is followed by metabolic barrier reduction, innate immune tuning, and checkpoint blockade or T-cell recovery, ultimately aiming to generate a hotter tumor immune microenvironment, improve chemosensitization, and reduce relapse.

A practical combination framework is shown in **Figure 5**. In OXPHOS-high residual disease, rational combinations may include chemotherapy with short-course OXPHOS or mitochondrial translation targeting. In lactate-dominant immune-cold states, combinations may prioritize LDHA/MCT inhibition or acidity-focused approaches. In hypoxic-adenosinergic tumors, oxygen-consumption reduction may be paired with adenosine pathway blockade. In mtDNA-STING-low cold tumors, controlled innate immune activation may be appropriate, whereas mtDNA-STING-high myeloid-suppressive tumors may require suppression of chronic myeloid capture or active myeloid reprogramming. The central concept is to convert mitochondrial stress from a survival signal into a coordinated immunologic alarm.

## Discussion

The conceptual contribution of this review is not to claim that mitochondrial rewiring universally and directly explains every form of chemoresistance or immune coldness. Rather, it adopts a more discriminating framework in which mitochondria can act as direct effectors, ecological amplifiers, or markers of a shared post-therapy selection process. This distinction is crucial because the same observed phenotype-an OXPHOS-high residual lesion with poor T-cell function-may arise from very different underlying mechanisms in different tumors.

This framework also reframes what is meant by “immune cold.” In practice, immune desert, immune excluded, and immune suppressed tumors are not metabolically interchangeable (15, 16). Some mitochondrial outputs may primarily impede entry, others may disable priming, and still others may convert infiltrating T cells into terminally exhausted cells. A useful model is therefore not a single causal arrow, but a mitochondrial immune set point: before treatment, each tumor has a baseline relationship among cancer-cell respiration, metabolite export, hypoxia, stromal architecture, antigenicity, innate sensing, and lymphocyte fitness. Chemotherapy perturbs this set point. In some tumors, cytotoxic injury transiently increases antigen release and danger signaling enough to attract and activate immunity. In others, chemotherapy selects OXPHOS-high persisters, increases lactate and myeloid stress signals, and leaves behind a colder immune topology than the baseline state.

Mechanism-specific evidence should also be weighed differently rather than discussed under one generic causal label. Lactate/acid stress and 2-HG provide the clearest examples of direct tumor-to-immune suppression, because defined transport, signaling, and metabolic routes link metabolite exposure to impaired effector immunity. mtDNA-STING signaling is also supported by strong experimental data, but its outcome is conditional and can shift from immunogenic to suppressive according to timing, source cell, compartmentalization, and the dominant responding myeloid or dendritic population. Hypoxia occupies an intermediate position: it can directly constrain T-cell function and promote adenosinergic suppression, yet it also frequently reflects shared vascular, stromal, and treatment-imposed selective pressures that simultaneously favor immune exclusion and mitochondrial adaptation. Succinate and fumarate remain the most context-dependent mechanisms in this framework, because source, concentration, receptor engagement, and spatial localization are difficult to infer *in vivo*, making direct immune suppression harder to prove consistently. Reverse causation is most plausible for OXPHOS-high residual disease and hypoxic immune exclusion, where reduced immune-cell competition may further stabilize respiratory tumor states, but is less convincing as a primary explanation for 2-HG or acute lactate/acid stress. This mechanism-by-mechanism grading is clinically important because different levels of evidence justify different levels of therapeutic confidence.

A further layer of persistence is epigenetic memory. Mitochondrial metabolites do not only exert acute signaling effects; they can also leave durable chromatin consequences in both tumor and immune cells. This is most evident in exhausted CD8+ T cells, where reinvigorated or antigen-cleared populations can retain stable epigenetic scars that constrain full functional recovery (99, 100). In parallel, repeated exposure to 2-HG, fumarate, succinate, redox stress, and intermittent hypoxia may stabilize transcriptional programs that outlast the initiating metabolic cue. This helps explain why some post-chemotherapy lesions remain immunologically cold even after a single suppressive metabolite is reduced: the niche may carry a memory of prior metabolic selection.

The same logic helps explain why some mitochondria-targeted therapies have disappointed. If OXPHOS inhibition is used without knowing whether the resistant compartment actually depends on OXPHOS, the result may be nonspecific toxicity. If lactate is blocked without restoring T-cell mitochondrial fitness or relieving stromal exclusion, the tumor may remain immune-excluded. If STING is activated in a niche dominated by suppressive myeloid cells, innate signaling may strengthen the wrong compartment. Mitochondria-targeted therapy should therefore be designed as ecological therapy: the endpoint is not only reduced tumor-cell ATP, but restoration of an immune state capable of clearing residual disease.

The field also needs better spatial and temporal measurements. Bulk RNA sequencing can identify OXPHOS signatures, but it cannot show whether high respiration is located in tumor cells, macrophages, Tregs, dendritic cells, or exhausted T cells. Metabolomics can measure lactate, succinate, fumarate, 2-HG, or itaconate, but it often loses the gradients and compartmentalization that determine immune meaning. Single-cell transcriptomics can classify immune states, but only indirectly estimates mitochondrial function. Recent studies have already begun to resolve this ecology with greater precision, including chemotherapy-induced myeloid spatial capture of exhausted T cells (49), macrophage-derived itaconate-driven resistance to anti-PD-1 therapy (51), mitochondrial control of dendritic-cell antitumor programs (52), and senescent-tumor-cell mtDNA-driven PMN-MDSC suppression (87). The next generation of studies should combine spatial transcriptomics, multiplex imaging, isotope tracing, oxygen mapping, mitochondrial activity reporters, and functional immune assays before and after chemotherapy. Without time-resolved spatial data, the immune-cold state will remain a static label rather than a targetable process.

Another unresolved issue is dose and timing. Mitochondrial stress can sensitize tumor cells, but immune cells also require mitochondria for proliferation, memory formation, dendritic-cell cross-priming, and cytotoxic persistence. A mitochondria-targeted agent given before chemotherapy may alter tumor oxygenation and apoptotic priming; given after chemotherapy, it may affect residual persisters or immune-cell recovery; given continuously, it may suppress both. This creates an opportunity for adaptive treatment schedules guided by early metabolic biomarkers rather than fixed dosing alone. In practice terms, short-course respiratory targeting is most plausible after chemotherapy has exposed antigen and selected a respiratory residual compartment; normalization of lactate, acidity, or adenosine may need to precede or accompany checkpoint blockade so that T cells encounter a less suppressive niche; and innate activators such as controlled STING stimulation are most likely to help when dendritic-cell competence is preserved and chronic myeloid capture is limited. The best regimen may therefore be intermittent, compartment-specific, biomarker-guided, and timed to the dominant immune-cold topology rather than applied continuously.

Patient heterogeneity deserves equal emphasis. The same mitochondria-immune circuit is unlikely to operate identically across metabolically healthy versus metabolically unhealthy obesity, insulin-sensitive versus insulin-resistant hosts, or male and female immune systems. Obesity can reshape nutrient partitioning within the tumor microenvironment and suppress CD8+ T-cell function (101), whereas sex is a biological variable that influences innate and adaptive immune responses across malignancy and therapy (102). These host-level variables are rarely incorporated into chemoresistance studies, yet they may alter baseline oxygen consumption, myeloid composition, lactate handling, endocrine signaling, and the probability that a residual lesion becomes immune-excluded rather than immune-suppressed.

Finally, the proposed model has direct clinical implications for patient selection. Tumors with high mitochondrial mass, OXPHOS signatures after chemotherapy, low T-cell infiltration, high lactate transport, hypoxia-adenosine activity, or suppressive mtDNA-STING myeloid programs may represent rational populations for mitochondria-metabolism-immunity combinations. Conversely, tumors with active immune infiltration but fragile T-cell mitochondrial fitness may require immune-cell support rather than aggressive respiratory blockade. The central clinical question is not simply whether mitochondria are active, but in which cell type, at which treatment phase, within which immune-cold topology, and with what immune consequence.

## Conclusions

Mitochondrial metabolic reprogramming offers a useful framework for linking chemotherapy resistance with immune-cold tumor evolution, but the relationship is context dependent rather than universally linear. By increasing OXPHOS dependence, buffering redox stress, remodeling mitochondrial quality control, exporting immunoregulatory metabolites, and reshaping innate immune sensing, tumor and myeloid mitochondria can help convert cytotoxic injury into residual-disease ecology.

Breaking this loop will require more than adding a mitochondrial inhibitor to chemotherapy. The next step is precision immunometabolic scheduling: identify the mitochondrial state of residual cancer cells, define the immune-cold topology and the suppressed immune compartment, and apply therapies that make mitochondrial stress visible to the immune system while preserving immune-cell fitness. If successful, targeting the mitochondria-immune interface could help transform chemoresistant immune-cold tumors into lesions that are both drug-sensitive and immunologically vulnerable.

## Glossary

2-HG: 2-hydroxyglutarate
ABC: ATP-binding cassette
alpha-KG: alpha-ketoglutarate
AML: acute myeloid leukemia
AMPK: AMP-activated protein kinase
ATP: adenosine triphosphate
CAFs: cancer-associated fibroblasts
cDC1: conventional type 1 dendritic cell
cGAS: cyclic GMP-AMP synthase
CSCs: cancer stem-like cells
CTLs: cytotoxic T lymphocytes
DAMPs: damage-associated molecular patterns
DCs: dendritic cells
ETC: electron transport chain
FAO: fatty acid oxidation
FH: fumarate hydratase
HIF-1alpha: hypoxia-inducible factor-1alpha
ICD: immunogenic cell death
ICB: immune checkpoint blockade
IDH: isocitrate dehydrogenase
IFN: interferon
IL: interleukin
LDHA: lactate dehydrogenase A
MCT: monocarboxylate transporter
MDSCs: myeloid-derived suppressor cells
MIMP: mitochondrial inner membrane permeabilisation
MOMP: mitochondrial outer membrane permeabilization
mtDNA: mitochondrial DNA
mtROS: mitochondrial reactive oxygen species
NK: natural killer
OXPHOS: oxidative phosphorylation
PGC-1alpha: peroxisome proliferator-activated receptor-gamma coactivator-1alpha
PINK1: PTEN-induced kinase 1
PMN-MDSCs: polymorphonuclear myeloid-derived suppressor cells
ROS: reactive oxygen species
SDH: succinate dehydrogenase
STING: stimulator of interferon genes
TAMs: tumor-associated macrophages
TCA: tricarboxylic acid
TCF1: T cell factor 1
TGF-beta: transforming growth factor-beta
TIME: tumor immune microenvironment
TME: tumor microenvironment
TOX: thymocyte selection-associated high mobility group box
Tregs: regulatory T cells
VDAC: voltage-dependent anion channel.
